# A long-lived pool of PINK1 imparts a molecular memory of depolarization-induced activity

**DOI:** 10.1126/sciadv.adr1938

**Published:** 2025-02-28

**Authors:** Liam Pollock, Ioanna Ch. Georgiou, Emma V. Rusilowicz-Jones, Michael J. Clague, Sylvie Urbé

**Affiliations:** ^1^ Biochemistry, Cell and Systems Biology, Institute of Systems, Molecular and Integrative Biology, University of Liverpool, Crown St., Liverpool L69 3BX, UK.; ^2^ Center for Molecular Biology of Heidelberg University (ZMBH), DKFZ-ZMBH-Alliance, 69120 Heidelberg, Germany.

## Abstract

The Parkinson’s disease–linked kinase, PINK1, is a short-lived protein that undergoes cleavage upon mitochondrial import leading to its proteasomal degradation. Under depolarizing conditions, it accumulates on mitochondria where it becomes activated, phosphorylating both ubiquitin and the ubiquitin E3 ligase Parkin, at Ser^65^. Our experiments reveal that in retinal pigment epithelial cells, only a fraction of PINK1 becomes stabilized after depolarization by electron transport chain inhibitors. Furthermore, the observed accrual of PINK1 cannot be completely accounted for without an accompanying increase in biosynthesis. We have used a ubiquitylation inhibitor TAK-243 to accumulate cleaved PINK1. Under these conditions, generation of unconjugated “free” phospho-ubiquitin serves as a proxy readout for PINK1 activity. This has enabled us to find a preconditioning phenomenon, whereby an initial depolarizing treatment leaves a residual pool of active PINK1 that remains competent to seed the activation of nascent cleaved PINK1 following a 16-hour recovery period.

## Teaser

Mitochondrial stress activates a long-lived pool of PINK1 that retains activity long after mitochondria have recovered.

## INTRODUCTION

PINK1 is a Parkinson’s disease–linked kinase that selectively accumulates at impaired or damaged mitochondria (*1*–*3*). Here, it phosphorylates Ser^65^ on both ubiquitin (hereafter pUb) and the E3 ubiquitin ligase Parkin (PRKN) (*4*–*8*). This activates Parkin, sparking a feed-forward cascade that results in widespread ubiquitylation at the mitochondrial surface, leading to mitophagy (*9*, *10*). The accepted model suggests that PINK1 is constitutively expressed and imported into mitochondria via the outer mitochondrial import (TOM) complex. It then engages with the TIM17/TIM23 inner mitochondrial membrane (IMM) translocase complex and undergoes cleavage within the mitochondrial matrix by the matrix mitochondrial processing peptidase (MPP) and within its transmembrane domain by the inner mitochondrial membrane presenilin associated rhomboid-like (PARL) protease (*1*, *11*, *12*). The cleaved form of PINK1 (cPINK1) is released from the mitochondria and rapidly degraded by the proteasome (*13*, *14*). Loss of the mitochondrial membrane potential prompts uncleaved membrane anchored PINK1 to accumulate in association with the TOM complex on the outer mitochondrial membrane, with the kinase domain facing the cytosol (*15*–*20*). This PINK1 stabilization requires the small TOM complex subunit TOM7 (*21*, *22*).

In the presence of proteasome inhibitors, cPINK1 accumulates in the cytosol (*13*). N-terminal truncated forms of PINK1 mimic this cleavage and show residual kinase activity toward ubiquitin (*23*). Moreover, the kinase activity of truncated PINK1 has been reported to protect neurons from the neurotoxin MPTP (*24*) and can inhibit global translation via phosphorylation of eEF1A (*25*). The influence of PINK1 kinase activity extends to the nucleus, and several proteins involved in nuclear confined processes acquire pSer^65^ ubiquitylation following mitochondrial depolarization in a PINK1-dependent manner (*26*–*29*). Whether or not these modifications require a freely diffusible form of PINK1 is currently unclear. While accumulation of full-length PINK1 at mitochondria provides a sensor for mitochondrial import stress, the accumulation of cytosolic cleaved PINK1 may provide an analogous sensing mechanism for proteasomal stress. Concordantly the truncated form of PINK1 can protect against cell death induced by proteasome inhibition (*30*, *31*).

In neurons, the transit to and residence time within synaptic terminals far exceeds the lifetime of PINK1. To preserve PINK1-dependent quality control at nerve terminals, PINK1 mRNA attaches to the mitochondrial outer membrane protein synaptojanin 2–binding protein via synaptojanin 2 (*32*). Furthermore, PINK1 has been linked to the TOM20-dependent mitochondrial localization and Parkin-mediated translational derepression of discrete nuclear encoded respiratory chain complex proteins (*33*).

Here, we have used TAK-243, an inhibitor of the E1 ubiquitin activating enzyme UBA1, to accumulate cPINK1 in an RPE1 cell line that naturally lacks Parkin (*34*, *35*). We find that cPINK1 continues to be generated under mitochondrial depolarizing conditions while a pathway to full-length PINK1 is concurrently activated. Our data suggest that this accumulation of full-length PINK1 cannot be fully accounted for by stabilization of PINK1 alone and likely reflects an element of increased translation. TAK-243 application, during recovery from a mitochondria depolarizing treatment, allows us to compare the rate at which conjugated pUb is lost alongside total ubiquitin. Notably, we find a second wave of free pUb generation, coincident with the emergence of cPINK1. On investigating recovery of cells from mitochondrial depolarization, we find that a prior depolarizing treatment elicits phosphorylation of ubiquitin and of cPINK1 generated in response to TAK-243 16 hours after removal of the depolarizing agents. We propose a model in which (i) PINK1 accumulation, under depolarizing conditions, in part reflects mobilization of its own translation at mitochondria, and (ii) an unexpectedly stable residual pool of PINK1 maintains an activity on reenergized mitochondria that can be directed toward activation of nascent PINK1 protein.

## RESULTS

### TAK-243 redirects PINK1 activity toward free ubiquitin

**Fig. 1.**
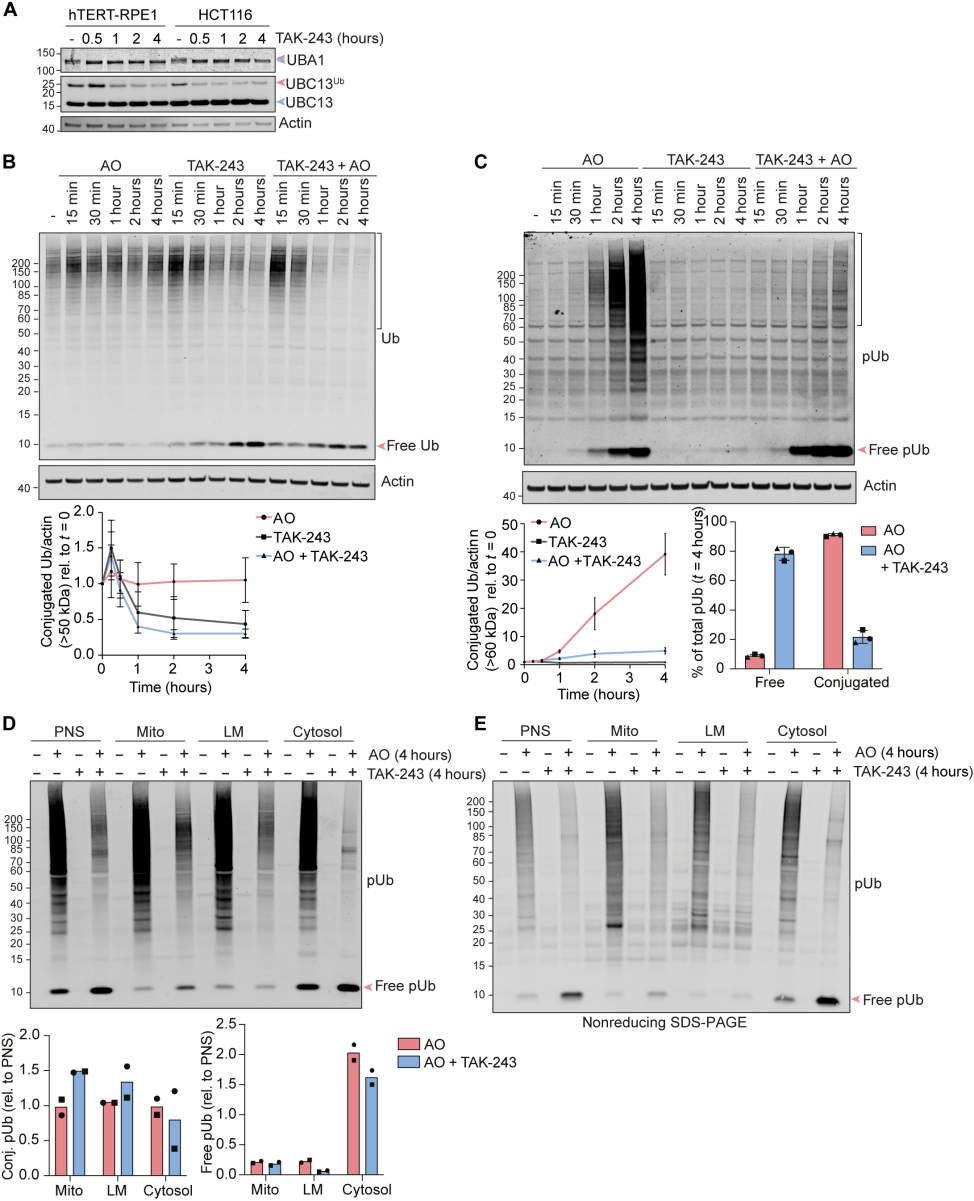
The UBA1 inhibitor TAK-243 reveals de novo phosphorylation of free ubiquitin in the cytosol. (**A**) hTERT-RPE1 and HCT116 cells were treated ± TAK-243 (1 μM) for the indicated times, lysed, and proteins analyzed by nonreducing SDS-PAGE and Western blot. Blue and red arrowheads indicate the unloaded forms and the Ub-loaded forms, respectively, of UBA1 and UBC13. Representative Western blot of two independent experiments. (**B** and **C**) hTERT-RPE1 cells were treated ± TAK-243 (1 μM), antimycin A (1 μM) and oligomycin A (1 μM) (AO), or both TAK-243 and AO. Representative Western blot of three independent experiments and quantification showing the decay of conjugated ubiquitin (B) and accumulation of free and conjugated pSer65-Ubiquitin [pUb (C)]. Brackets indicate the molecular weight (MW) range used for the quantification. Error bars show SD. (**D** and **E**) hTERT-RPE1 cells were treated for 4 hours ± TAK-243 (1 μM), AO (1 μM), or TAK-243 and AO and subjected to subcellular fractionation. PNS, post-nuclear supernatant; Mito, mitochondria-enriched fraction; LM, light membrane fraction. SDS-PAGE was performed under reducing (D) or nonreducing (E) conditions to assess levels of free pUb. Bracket indicates the MW range of signal used for conjugated pUb quantification. Quantification of data shown in (D) shows the enrichment of conjugated and free pUb in mitochondrial, LM, and cytosolic fractions (normalized to PNS) for two independent experiments.

We have used RPE1 cells that lack detectable Parkin but still present a pool of mitochondrial ubiquitylation that serves as PINK1 substrate (*35*), which is likely to reflect the activity of other resident mitochondrial E3 ligases such as MARCH5 or MUL1 (*36*–*39*). Throughout this study, we have used TAK-243 to inhibit the ubiquitin E1 enzyme UBA1, the principal initiating enzyme of ubiquitylation cascades (*34*, *40*, *41*). Its application results in the rapid downshift in molecular weight of UBA1 under nonreducing conditions, consistent with a loss of charged ubiquitin in HCT116 and hTERT-RPE1 cells. A more gradual loss of ubiquitin from the E2 enzyme UBC13 (UBE2N) is also observed (Fig. 1A). Acute application of TAK-243 can be used to visualize the dissipation of global ubiquitylation within hours in RPE1 cells (Fig. 1B). We compared the pUb Western blotting profile of cells subjected to mitochondrial depolarization by the application of the respiratory chain inhibitors antimycin A and oligomycin A (AO), in the presence and absence of TAK-243. Existing mitochondrial ubiquitin can serve as a PINK1 substrate, but the pUb signal is highly amplified by depolarization-induced ubiquitylation even though these cells do not express Parkin (Fig. 1C). In the presence of TAK-243, the complexity of the pUb profile following mitochondrial depolarization is reduced. A few higher molecular weight bands become more prominent against this reduced background and are candidates for “priming” substrates that enable the subsequent cascade (*39*). Notably, when ubiquitylation is inhibited, PINK1 directs its activity toward free ubiquitin molecules (Fig. 1, C to E). The distribution of pUb within subcellular fractions is shown in Fig. 1 (D and E). pUb conjugation is clearly not restricted to mitochondria, and the population of free pUb is highly enriched in the cytosolic fraction. SDS–polyacrylamide gel electrophoresis (SDS-PAGE) is routinely carried out under reducing conditions, which liberates E1- and E2-associated ubiquitin as well as any otherwise thioester-linked ubiquitin. Under nonreducing conditions, we retain free pUb, confirming that this is part of the free ubiquitin pool rather than loaded onto conjugating enzymes (Fig. 1E). Note that most of this “free” pUb is unaffected by conditions that inactivate deubiquitylase (DUB) activity, namely inclusion of *N*-ethylmaleimide (NEM) in our NP40 lysis buffer (fig. S1A) or direct lysis in super-heated (110°C) SDS buffer (fig. S1B).

### TAK-243 treatment stabilizes cPINK1

**Fig. 2.**
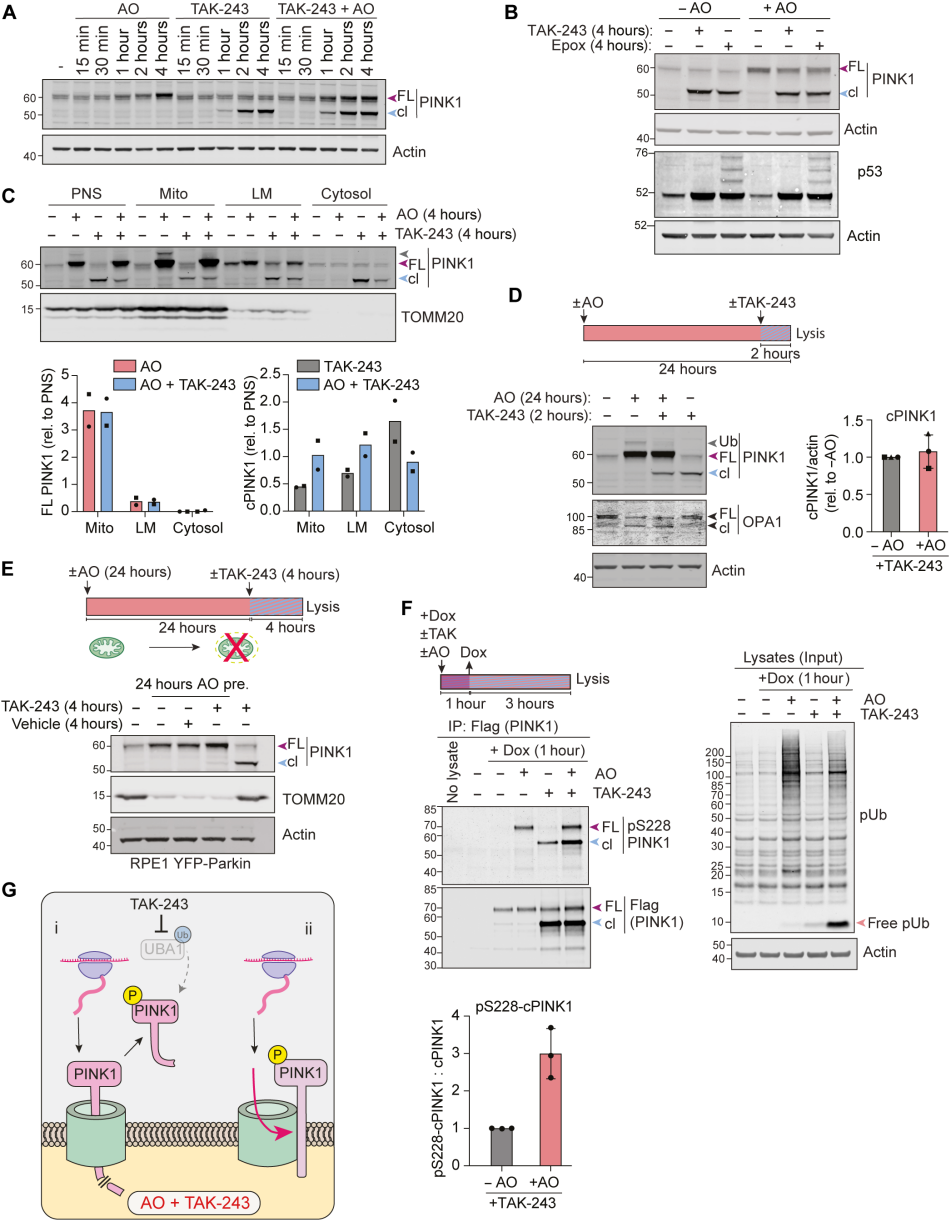
TAK-243 reveals continued processing of PINK1 and generation of cleaved active PINK1 at depolarized mitochondria. (**A** and **B**) hTERT-RPE1 cells were treated ± TAK-243 (1 μM), antimycin A (1 μM) and oligomycin A (1 μM) (AO), or ± epoxomicin (100 nM) as indicated. Representative Western blots of three independent experiments. Colored arrowheads correspond to full-length (FL) and cleaved PINK1 (cl). (**C**) hTERT-RPE1 cells were treated ± AO, ±TAK-243 for 4 hours before subcellular fractionation (the same samples as in Fig. 1D). Representative Western blot and associated quantification. (**D**) hTERT-RPE1 cells were pretreated ± AO for 22 hours before addition of TAK-243 for a further 2 hours. Representative Western blot and quantification of cleaved PINK1. Error bars, SD; *n* = 3 independent experiments. (**E**) hTERT-RPE1 YFP-Parkin cells were pretreated ± AO for 24 hours before addition of TAK-243 for a further 4 hours. Representative Western blot of four independent experiments. (**F**) hTERT-RPE1 PINK1 KO cells inducibly expressing PINK1-Flag were treated with doxycycline (Dox, 0.1 μg/ml) for 1 hour ± AO, ±TAK-243, and lysates subjected to anti-Flag immunoprecipitation (IP). Representative Western blot and quantification. Error bars, SD; *n* = 3 independent experiments. (**G**) Working model proposing two distinct pools of PINK1 on depolarized mitochondria: (i) A labile pool is imported, processed, and released as cPINK1 from mitochondria and can be visualized by TAK-243 treatment; (ii) a separate pool of PINK1 remains intact and accumulates upon depolarization. Both pools are active in this model.

The application of TAK-243 can be functionally equivalent to proteasome inhibition in terms of protein stabilization. We compared the effects of treatment with AO, TAK-243, or the proteasome inhibitor epoxomicin, alone or in combination. Here, we confirm that TAK-243 leads to the appearance of cPINK1, exactly as previously reported with proteasome inhibitors (Fig. 2A) (*3*, *13*, *42*). Both epoxomicin and TAK-243 generate similar amounts of cPINK1 (Fig. 2B). The accumulation rate of this cPINK1 is unaffected by concomitant AO treatment, which instead leads to the parallel acquisition of full-length PINK1. The full-length PINK1 that accumulates under depolarizing conditions is isolated from this cleavage pathway and accrues at the same rate ± UBA1 inhibition (Fig. 2A). As expected, full-length PINK1 is confined to mitochondria, while a substantial fraction of cPINK1 appears in the cytosolic fraction (Fig. 2C). We next accumulated full-length PINK1 through a long period of depolarization (24 hours) and added TAK-243 for the final 2 hours. cPINK1 is generated upon TAK-243 application with or without mitochondrial depolarization (Fig. 2D). Note that this experiment also reveals a minor fraction of mono-ubiquitylated full-length PINK1, which is lost within the 2 hours of TAK-243 treatment. To demonstrate that the TAK-243–induced cPINK1 requires mitochondrial processing, we used a method to remove mitochondria from cells in advance of drug application (*35*, *43*). RPE1 cells overexpressing yellow fluorescent protein (YFP)–Parkin are first treated with AO for 24 hours, which removes mitochondria through mitophagy. In these mitochondria-deficient cells, cPINK1 no longer accumulates following TAK-243 application (Fig. 2E).

Phosphorylation at Ser^228^ provides a signature for PINK1 activation (*23*). To determine whether cPINK1 occurs in an activated form, we turned to an engineered RPE1 Flp-In cell line, in which endogenous PINK1 has been knocked out and replaced with an inducible PINK1-Flag transgene (fig. S2). This enabled us to immunoprecipitate PINK1-Flag and to demonstrate its activity status using a pSer^228^-PINK1–directed antibody as a proxy reporter (*23*). The level of cPINK1 accumulated upon TAK-243 treatment is independent of AO treatment, but its activation is strongly enhanced by such depolarization (Fig. 2F). Together, these data suggest the coexistence of two distinct fates for PINK1, with one pool undergoing constitutive cleavage irrespective of the imposed decline in membrane potential, while a second accumulates as a full-length species only upon this depolarization (Fig. 2G).

### PINK1 stability measurements

**Fig. 3.**
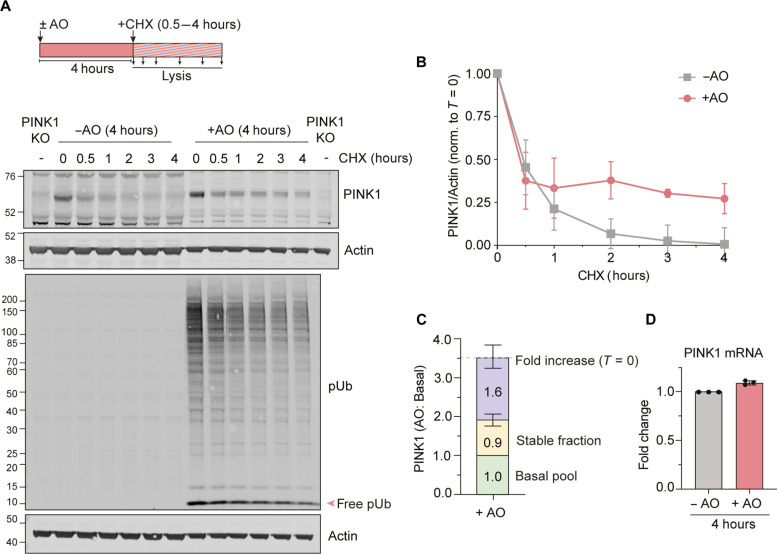
PINK1 stability measurements. (**A**) hTERT-RPE1 cells were treated for 4 hours with AO (1 μM) followed by addition of cycloheximide (CHX; 100 μg/ml, ± AO) for 0.5 to 4 hours. Experimental configuration and representative Western blots are shown. Protein loading was adjusted to obtain a similar PINK1 signal at *T* = 0 (3× protein loaded for the first seven lanes in the PINK1 blot; 2× for the first seven lanes of the pUb blot). An untreated PINK1 KO cell–derived sample was loaded alongside for background subtraction. (**B**) Quantification of the rate of PINK1 decay assessed as in (A). Error bars show SD, *n* = 4 independent experiments. (**C**) Graph showing the fold increase in PINK1 levels after 4 hours of AO treatment [*T* = 0 hours in (A)] and the fraction of the accrued PINK1 that is stable [PINK1 (+AO at *T* = 3 hours)/PINK1 (+AO at *T* = 0 hours)], leaving an unaccounted for pool that may reflect increased translation. Error bars (fold increase and stable fraction) show SD of four independent experiments. (**D**) Quantitative RT-PCR reactions of PINK1 (normalized to actin) were performed with cDNA derived from cells treated ± AO (1 μM) for 4 hours. Error bars show SD, *n* = 3 independent experiments.

Why does full-length PINK1 accumulate under depolarizing conditions if the pathway leading to cPINK1 remains fully intact? Previous studies in Parkin overexpressing cells have suggested that this full-length PINK1 accumulates in a stable form when cells are treated with the protonophore carbonyl cyanide m-chlorophenylhydrazone (CCCP) (*3*). Using a cycloheximide chase protocol, we have compared the half-life of full-length PINK1 in untreated cells versus cells that have been treated for 4 hours with AO (Fig. 3A). We take advantage of the high linearity and dynamic range of the LICOR Odyssey infrared scanner for quantitation. For these experiments, we have also been careful in our choice of antibody, making sure that we can clearly discriminate a specific bona fide full-length PINK1 band that is absent in isogenic PINK1 KO cells (fig. S3 and table S1).

For untreated cells, the half-life of PINK1 revealed by cycloheximide chase is ~30 min (Fig. 3B). To be at steady state, this degradation rate must be exactly balanced by the rate of protein synthesis. After 4 hours of AO treatment, PINK1 levels have increased ~3.5-fold (Fig. 3C), while mitochondrial membrane potential was substantively lost (fig. S4). However, only ~25% of this PINK1 is stable (Fig. 3, B and C), with the remainder showing a fast decay rate similar to untreated cells. Under these conditions, the pUb also declines in step with PINK1 levels (Fig. 3A). PINK1 mRNA levels are unchanged upon depolarization (Fig. 3D). Other PINK1 antibodies we have trialed gave a higher background signal (fig. S3 and table S1), but nevertheless recapitulate our identification of a fast decay pool (fig. S5).

### AO preconditioning of PINK1 activity

**Fig. 4.**
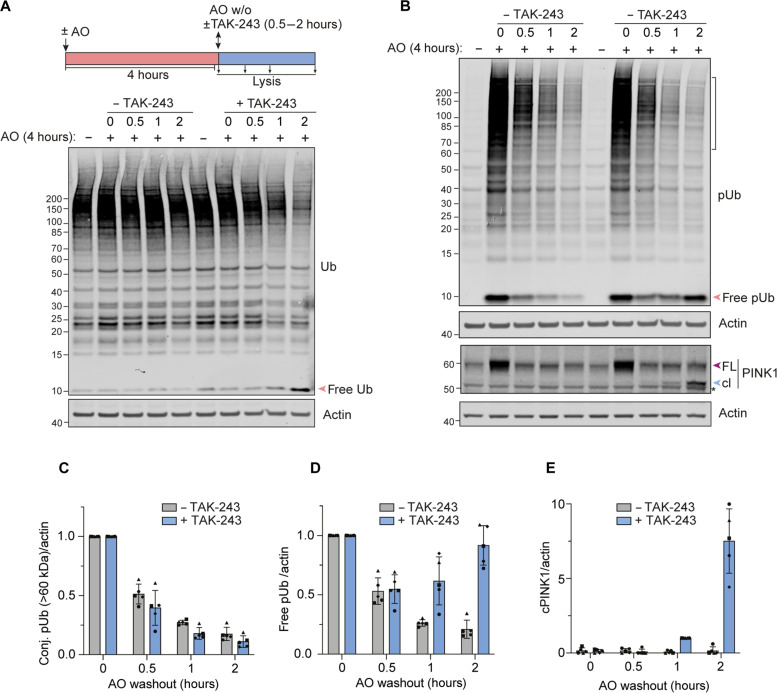
Correlation between cleaved PINK1 and free pUb generation upon E1 inhibition. (**A**) Schematic diagram showing experimental overview and representative Western blot for ubiquitin. hTERT-RPE1 cells were treated ± AO (1 μM) for 4 hours, followed by lysis (0 hour) or replacement of media (washout) and a 0.5 to 2 hours chase ± TAK-243 (1 μM). (**B**) Representative Western blots for pUb and PINK1 from experiments depicted schematically in (A). The bracket indicates the range used for conjugated pUb quantification. * indicates a nonspecific band. (**C** to **E**) Quantification of data represented in (B) showing conjugated pUb (conj. pUb) decay (C), accumulation of free pUb (D), and cleaved PINK1 (cPINK1) (E). Error bars show SD for five independent experiments.

We next conducted a series of experiments where we first subjected cells to a 4-hour treatment with AO and then washed out these drugs leading to reestablishment of the mitochondrial membrane potential (fig. S4). Firstly, TAK-243 was applied at the point of washout and showed no influence on the dissipation rate of conjugated pUb (Fig. 4, A to C). Free pUb levels also decayed, suggesting that this rate is likely dominated by phosphatase rather than DUB activity, in line with a previous report (*28*). However, in TAK-243–treated samples, we noticed a prominent second wave of free pUb generation, coincident with the emergence of cPINK1 (Fig. 4, B, D, and E).

**Fig. 5.**
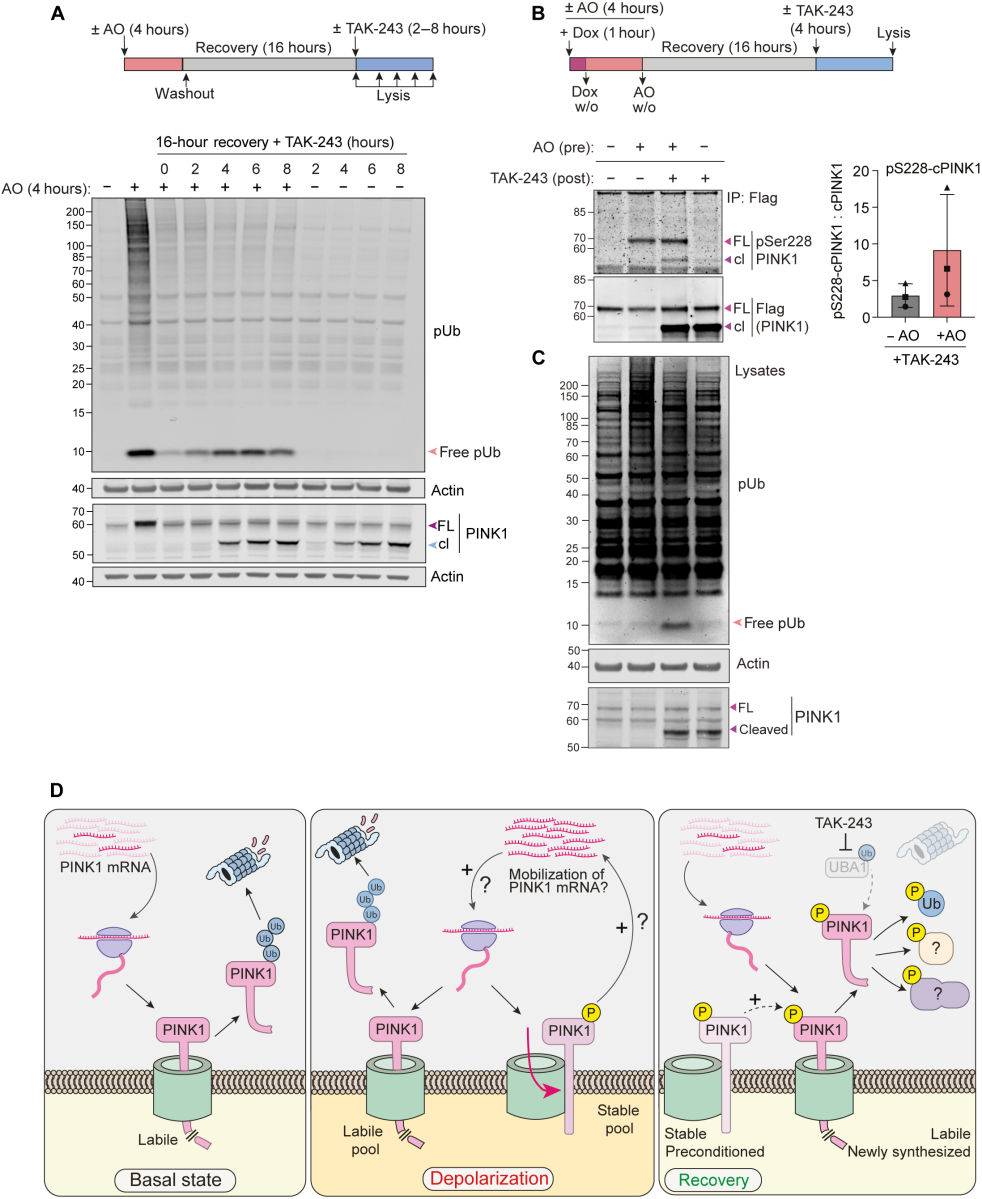
PINK1 exhibits sustained activity after mitochondria have recovered from depolarization. (**A**) hTERT-RPE1 cells were treated for 4 hours ± AO (1 μM) and lysed or incubated in media without AO (washout; w/o). After 16 hours of recovery, the cells were either lysed or treated with TAK-243 (1 μM) for indicated times. Representative of three independent experiments. (**B** and **C**) hTERT-RPE1 PINK1 KO cells inducibly expressing PINK1-FLAG were cotreated with doxycycline (Dox) ± AO for 1 hour, washed, and incubated ± AO for further 3 hours. AO was washed out and cells left to recover for 16 hours before treatment ± TAK-243 for 4 hours. Lysates were subjected to anti-Flag IP. Bound proteins (B) were analyzed alongside lysates (C) by Western blot. Graph shows pSer^228^-cPINK1 normalized to total cPINK1 generated upon TAK-243 treatment after recovery from a prior depolarization insult (AO). Error bars, SD; *n* = 3 independent experiments. (**D**) Working model of PINK1 behavior under basal, depolarizing, and recovery conditions. Under resting conditions (basal state), PINK1 is rapidly turned over through cleavage in mitochondria and subsequent ubiquitin-dependent degradation. This labile pool persists under depolarizing conditions alongside the emergence of a stable pool of active PINK1, which is proposed to promote the mobilization of PINK1 mRNA resulting in an increase in PINK1 protein synthesis. A small pool of PINK1 remains stable and active long after mitochondria have recovered from depolarization and can facilitate activation of nascent, labile PINK1, which can be visualized and stabilized by inhibiting ubiquitylation.

We then modified our protocol to include an extended recovery time (16 hours) before the application of TAK-243. The AO-enabled activation of freshly generated PINK1 (i.e. cPINK1) is retained during this extended recovery period, although the mitochondria fully recover their membrane potential within 2 hours (Fig. 5A and fig. S4). We next turned to our doxycycline-inducible PINK1-Flag cell model to examine the activation status of PINK1. We again administered TAK-243 following a 16-hour recovery period from a preceding depolarization event (Fig. 5, B and C). As expected, we see evidence of cPINK1 activation, which is dependent on the prior mitochondrial depolarization. A residual fraction of phosphorylated full-length PINK1 is detectable, which we propose acts as a seed for transphosphorylation of nascent PINK1 that is here accumulating as cPINK1 (Fig. 5D). This is also consistent with the lingering shadow of a slightly upshifted endogenous PINK1 band, likely to be phosphorylated PINK1 that is maintained over the recovery period from AO treatment (Fig. 5A, see lanes 3 to 7). These experiments reveal how such memory of a past event may be cached.

## DISCUSSION

Acute application of the ubiquitin E1 enzyme inhibitor TAK-243 has allowed us to visualize the generation of cPINK1 (*34*). The same product has previously been reported upon proteasome inhibition (*3*, *13*, *42*, *44*). TAK-243 treatment now offers an alternative method to generate this proteoform, with a divergent impact on the global distribution of ubiquitin. Both approaches provide an otherwise inaccessible glimpse of the constitutive mitochondrial cleavage rate of PINK1. Our finding that this rate is independent of our chosen depolarization conditions is unexpected given previous findings in HeLa cell models overexpressing Parkin (*3*). The canonical model proposes that once the mitochondrial targeting sequence of PINK1 has passed into the mitochondrial matrix, via the voltage-gated TIM23 complex, it is cleaved, enabling further PARL-mediated cleavage of the PINK1 transmembrane domain (*45*). Voltage-independent turnover of PINK1 is not unprecedented and PINK1 accumulation can occur on fully polarised mitochondria. PINK1 is stabilised when misfolded proteins accumulate in the matrix and under mitochondrial import stress (*46*, *47*). Experiments in HeLa cells lacking the small TOM complex subunit, TOM7, have revealed a compensatory cleavage under depolarizing conditions, which is thought to be mediated by the depolarization activated IMM protease OMA1 (*22*, *48*). It is suggested that TOM7 protects PINK1 from exposure to OMA1. Akabane *et al.* (*49*) have also noted that protonophore-induced stabilization of PINK1 is inhibited by coincident activation of protein kinase A, once again demonstrating efficient processing in the absence of a mitochondrial membrane potential.

How can we account for the observed accrual of full-length PINK1 following depolarization? We suggest that there are two populations of newly synthesized PINK1, one which follows a voltage-insensitive pathway leading to degradation, and a second voltage-sensitive pathway yielding a stable pool of full-length PINK1 (Fig. 2G). We propose that all PINK1 interacts with a TOM complex for import but that fates can differ. This partitioning could arise as a result of kinetic competition in folding or import steps, availability of cofactors, or spatial restrictions relative to other inner/outer mitochondrial membrane components.

In our adopted RPE1 cell model, this stable fraction cannot account for the full increase we observe in PINK1 upon depolarization by AO (Fig. 3C). We arrive at this conclusion after careful background subtraction and quantitation across multiple experiments using an instrument with a high linear dynamic range. Our knockout (KO) cell line provides an important control for antibody specificity and identification of background bands. Since we, and others, have also excluded an increase in PINK1 mRNA (Fig. 3D) (*3*), we propose that PINK1 translation must increase under these depolarizing conditions. Previous global proteomic studies have used the same logic to indicate the importance of differing translation efficiencies in determining protein abundance (*50*). PINK1 mRNA is known to bind to mitochondrial membranes in neurons (*32*). Similarly, some respiratory chain component mRNAs can be recruited to mitochondria and are derepressed in a PINK1-dependent manner (*33*). The second effect of mitochondrial depolarization is activation of PINK1 (*4*, *17*, *18*, *23*, *51*). A simple speculative model posits that depolarization leads to activation of the small fraction of preexisting full-length PINK1. This then acts to mobilize mitochondrial bound PINK1 mRNA for additional translation, which underlies the accrual of protein (Fig. 5D).

Our results show that the cPINK1, which accumulates upon proteasomal or E1 enzyme inhibition, contains an activated population. Under TAK-243 treatment, this activity manifests as an increase in the free pUb pool, but under other conditions of proteasomal stress, its ability to freely diffuse will open up a distinct palette of potential ubiquitylated substrates. pUb-modified proteins generated by PINK1 have been linked to nuclear processes including DNA repair (*28*). In principle, free pUb can be generated by DUB activity toward substrate proteins, but this is highly unlikely to be the predominant mechanism in our experiments. First of all, pUb chains are relatively poor DUB substrates (*52*, *53*), and, secondly, the relevant signal arises under conditions where the pool of conjugated ubiquitin has been depleted (TAK-243). While phosphorylation of ubiquitin and Parkin are the critical outputs for PINK1 governance of mitophagy, other substrates may be physiologically relevant (*29*). This viewpoint is supported by various studies indicating roles for PINK1 in translational control, mitochondrial calcium handling, and immune signaling (*27*, *54*–*56*).

Ischemic preconditioning imparts tissue protection in multiple organs. This is a process whereby a short nonlethal ischemia reperfusion protects against a subsequent severe ischemia-reperfusion injury (*57*). An intriguing study in the mouse kidney has identified mitophagy as an important mediator of this response. Moreover, following the first insult, PINK1 is up-regulated and depletion of PINK1 leads to a loss of protection (*58*). Here, we have found a conditioning phenomenon directly linked to PINK1 activity. Treatment with AO conditions the response to subsequent application of TAK-243 many hours later. This leads to activation of nascent cleaved PINK1 and phosphorylation of free ubiquitin. Although this accumulation of free pUb is epiphenomenological, it provides a convenient proxy for PINK1 activity. While this particular experimental condition has allowed us to uncover this behavior, we think that the underlying mechanism may be relevant to pathological settings that involve sequential insults to mitochondria. Our working model conceives that the medium for this “molecular memory” is provided by a small long-lived pool of activated PINK1 that primes the subsequent response (Fig. 5D).

## MATERIALS AND METHODS

### Cell culture

hTERT-RPE1 Flp-In TREX (a gift from J. Pines, London, UK; referred to as hTERT-RPE1) and hTERT-RPE1–YFP-PARKIN cells (a gift from J. Lane, Bristol, UK) were cultured in Dulbecco’s modified Eagle’s medium–F12 supplemented with 10% fetal bovine serum (FBS). hTERT-RPE1 Flp-In TREX cells expressing PINK1-3xFlag, referred to as hTERT-RPE1 PINK1 KO (PINK1-Flag), were maintained under blasticidin (10 μg/ml) and G418 (400 μg/ml) selection and induced with doxycycline (0.1 μg/ml). HCT116 cells were cultured in McCoy’s media supplemented with 10% FBS.

### Generation of PINK1 KO cells

PINK1 KO cells were generated using CRISPR-Cas9 with a PINK1-targeting sgRNA (5′- CACCGTACCCAGAAAAGCAAGCCG-3′) cloned into pU6-(BbsI)-CBh-Cas9-T2A-mCherry (Addgene, #64324). This plasmid was transfected into hTERT-RPE1 Flp-In TREX cells followed 24 hours later by fluorescence-activated cell sorting to select for mCherry-positive cells. Individual clones were isolated by single-cell dilution and validated by Western blotting and genomic DNA sequencing. Clone 6 harbored a single base deletion in both alleles (G130 in the coding sequence), resulting in a frameshift and early stop codon. This clone is referred to as PINK1 KO and was used for further generation of PINK1-Flag–expressing cells.

### Generation of an inducible hTERT-RPE1 PINK1 KO (PINK1-Flag) cell line

C-terminally tagged PINK1-3xFLAG (PINK1-Flag) was cloned into pcDNA5 FRTTO NeoR by Gibson assembly and transfected into hTERT-RPE1 Flp-In TREX PINK1 KO cells (hTERT-RPE1 PINK1 KO) alongside pOG44 (1:9). Two days after transfection, the cells were transferred to blasticidin (10 μg/ml) and G418 (400 μg/ml)–containing media and grown under selection for 2 weeks. Individual clones were isolated by single-cell dilution and validated by immunofluorescence and Western blot analysis. PINK1-Flag expression was induced with doxycycline (0.1 μg/ml) as indicated.

### Antibodies and reagents

Antibodies and other reagents are as follows: anti-PINK1 D8G3 (rabbit, 6946, 1:1000, Cell Signaling Technology), anti-PINK1 (rabbit, 81991-4-RR, 1:1000, ProteinTech) (“PT-Rec”; figs. S3 and S5), anti-PINK1 (rabbit, 23274-1-AP, 1:1000, ProteinTech) (“PT-Poly”; figs. S3 and S5), anti-PINK1 (rabbit, BC100-494, 1:1000, Novus Biologicals) (“Novus”; fig. S3), anti–pSer228-PINK1 (rabbit, generous gift from M. Muqit, MRC PPU, Dundee, UK, 1:170), anti–pSer65-Ub E2J6T (rabbit, 62802, 1:1000, Cell Signaling Technology), anti-UBA1 (rabbit, ab34711, 1:1000, Abcam), anti-UBC13 (rabbit, ab25885, 1:1000, Abcam), anti-ubiquitin (mouse, VU101, 1:2000, LifeSensors), anti-p53 (mouse, sc-126, 1:1000, Santa Cruz Biotechnology), anti-OPA1 (rabbit, ab42364, 1:1000, Abcam), anti-Actin (mouse, 66009, 1:10,000, Proteintech), anti-TOMM20 (rabbit, 11802-1-AP, 1:1000, Proteintech), anti-Flag (goat, NB600-344, 1:1000, Novus), anti-Flag (mouse, M2, F3165, 1:1000, Sigma-Aldrich), cycloheximide (C7698, Sigma-Aldrich), oligomycin A (75351, Sigma-Aldrich), antimycin A (A8674, Sigma-Aldrich), doxycycline (D9891, Sigma-Aldrich), epoxomicin (324800, Sigma-Aldrich), TAK-243 (MLN7243, S8341, Selleckchem), tetramethylrhodamine ethyl ester (TMRE) (ab113852, Abcam), and Hoechst 33342 (62249, Thermo Fisher Scientific).

### Cell lysate preparation and Western blotting

Cells were lysed on ice in NP-40 lysis buffer [0.5% Nonidet P 40 Substitute (74385, Sigma-Aldrich), 25 mM tris-HCl (pH 7.5), 100 mM NaCl, and 50 mM NaF] supplemented with mammalian protease inhibitors (MPIs) (Sigma-Aldrich) and PhosSTOP (Roche) (+ 20 mM NEM; fig. S1A) or lysed in SDS-lysis buffer (2% SDS, 1 mM EDTA, and 50 mM NaF) at 110°C (fig. S1B). Proteins were resolved using 4 to 12% NuPage gels and transferred to 0.45 or 0.2 μm of nitrocellulose membranes. Membranes were blocked in either 5% milk, 5% bovine serum albumin, or 0.1% fish skin gelatin (Ub) and 0.1% Tween20 in tris-buffered saline (TBS) before primary antibody incubation in the same blocking solution. Western blot visualization was performed using IRdye 800CW and 680LT coupled secondary antibodies with an Odyssey infrared scanner (Li-COR, Biosciences). For quantitations, signal values were obtained from Image Studio software after subtracting the background signal.

### Subcellular fractionation

hTERT-RPE1 cells were washed with ice-cold phosphate-buffered saline twice, harvested by scraping, and centrifugation at 1000*g* for 2 min at 4°C. Cell pellets were washed with HIM buffer [200 mM mannitol, 70 mM sucrose, 1 mM EGTA, and 10 mM Hepes-NaOH (pH 7.4)], centrifuged again at 1000*g* for 5 min, and resuspended in HIM buffer supplemented with MPI (Sigma-Aldrich), PhosSTOP (Roche), and 50 mM 2-chloroacetamide (CAA; Sigma-Aldrich). Cells were mechanically disrupted by shearing through a syringe with a 27G needle. The resulting homogenate was cleared from nuclei and unbroken cells by centrifugation at 600*g* for 10 min to obtain a post-nuclear supernatant. A crude mitochondrial fraction (Mito) was obtained by centrifugation at 7000*g* for 15 min and the post-mitochondrial supernatant was further separated by centrifugation at 100,000*g* for 30 min into light membrane (LM) and cytosolic fractions. The mitochondrial and LM fractions were resuspended in HIM buffer supplemented with MPI, CAA, and PhosSTOP. Equal amounts of protein were loaded for each fraction.

### Immunoprecipitation

hTERT-RPE1 PINK1 KO (PINK1-Flag) cells were treated with doxycycline (0.1 μg/ml) to induce PINK1 expression and treated as indicated before lysis in SDS-lysis buffer (2% SDS, 1 mM EDTA, and 50 mM NaF) at 110°C. Lysates (700 to 3500 μg) were diluted fourfold in immunoprecipitation (IP) dilution buffer [2.5% Triton X-100, 12.5 mM tris (pH 7.5), and 187.5 mM NaCl] supplemented with MPI and PhosSTOP. Lysates were cleared by centrifugation and incubated overnight with 100 μl of Pierce Anti-DYKDDDDK magnetic agarose (A36797). The immunoprecipitates were washed three times with IP wash buffer (1 part SDS-lysis buffer, 4 parts IP dilution buffer), twice with TBS, and once with Millipore water. Bound proteins were eluted with 50 μl of 1.5× nonreducing sample buffer [4.5% SDS, 93.75 mM tris-HCl (pH 6.8), and 15% glycerol] at 95°C for 10 min and then mixed with reducing sample buffer and analyzed by SDS-PAGE.

### RNA isolation and quantitative reverse transcription PCR

RNA was extracted using the Qiagen RNA extraction kit (74106). cDNA was then generated using Thermo Fisher Scientific RevertAid H Minus reverse transcription (Thermo Fisher Scientific; 11541515) supplemented with RNasin (Promega; N2611), polymerase chain reaction (PCR) nucleotide mix (Promega; U144B), and oligo (dT) 15 Primer (Promega; C1101). Quantitative PCR were performed in triplicate using primers against PINK1 (5’-GGACGCTGTTCCTCGTTA-3′, 5’-ATCTGCGATCACCAGCCA-3′) and ACTB (Actin, 5’-CACCTTCTACAATGAGCTGCGTGTG-3′; 5’-ATAGCACAGCCTGGATAGCAACGTAC-3′), and iTaq Mastermix (Bio-Rad; 172-5171) in a Bio-Rad CFX Connect real-time system. Fold change was calculated using the 2^−ΔΔCt^ method.

### Mitochondrial membrane potential measurements

The cells were seeded into μ-dishes (Ibidi) and stained with 50 nM TMRE and Hoechst 33342 (0.5 μg/ml) for 20 min before imaging at 37°C and 5% CO2 using a Zeiss LSM900 microscope and a 40× LD C-apochromat 1.1 numerical aperture water immersion objective. Images were processed and analyzed using FIJI v2.0 for quantitation and Adobe photoshop for illustration. Graph shows the corrected total cell fluorescence [CTCF = integrated density – (area of selected cell × mean fluorescence of background readings); number of cells imaged: dimethyl sulfoxide, 49; 4 hours AO, 70; 2-hour recovery, 53; 16-hour recovery, 57].
