# Supplementary material for: A long-lived pool of PINK1 imparts a molecular memory of depolarization-induced activity
Source: Sci Adv. 2025 Feb 28;11(9):eadr1938. doi: 10.1126/sciadv.adr1938 (PMC11870087; doi:10.1126/sciadv.adr1938)
Supplement: Supplementary file 1 — Figs. S1 to S5 Table S1 [file sciadv.adr1938_sm.pdf]

Science Advances
AAAS

# Supplementary Materials for

## A long-lived pool of PINK1 imparts a molecular memory of depolarization-induced activity

Liam Pollock *et al.*

Corresponding author: Michael J. Clague, clague@liv.ac.uk; Sylvie Urbé, urbe@liv.ac.uk

*Sci. Adv.* **11**, eadr1938 (2025)
DOI: 10.1126/sciadv.adr1938

**This PDF file includes:**

Figs. S1 to S5
Table S1

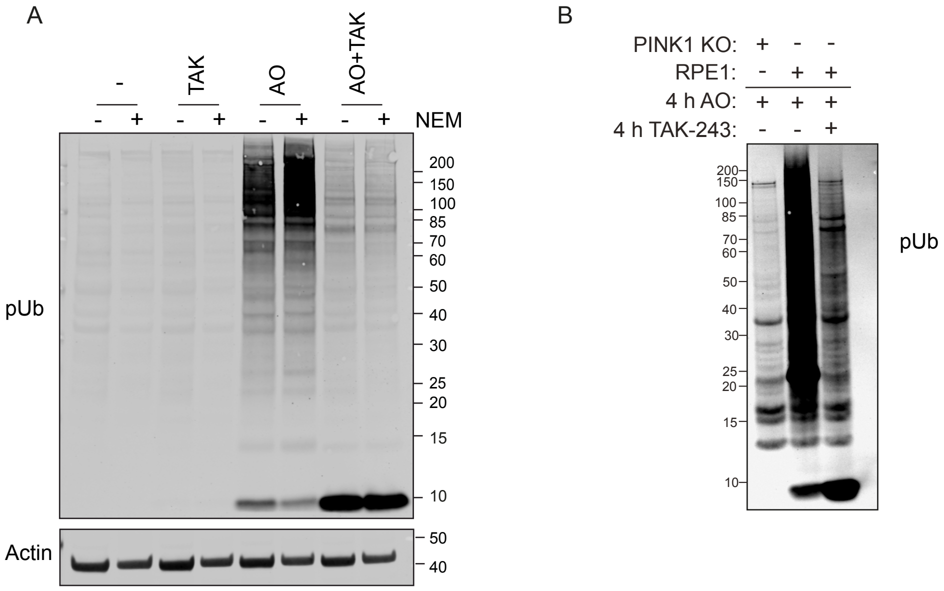


**Figure S1: Generation of unconjugated pUb in AO and TAK-243 treated cells**

**A.** hTERT-RPE1 cells were treated ± Antimycin A and Oligomycin A (AO, 1 µM each), ± TAK-243 (1 µM) for 4 h. Samples were lysed in NP40 lysis buffer ± 20 mM NEM and probed for pUb by western blotting. Representative western blot of two independent experiments.

**B.** hTERT-RPE1 and PINK1 KO cells were treated for 4 h with AO ± TAK-243 and lysed in superheated SDS-lysis buffer and probed probed for pUb by western blotting. Representative western blot of two independent experiments.

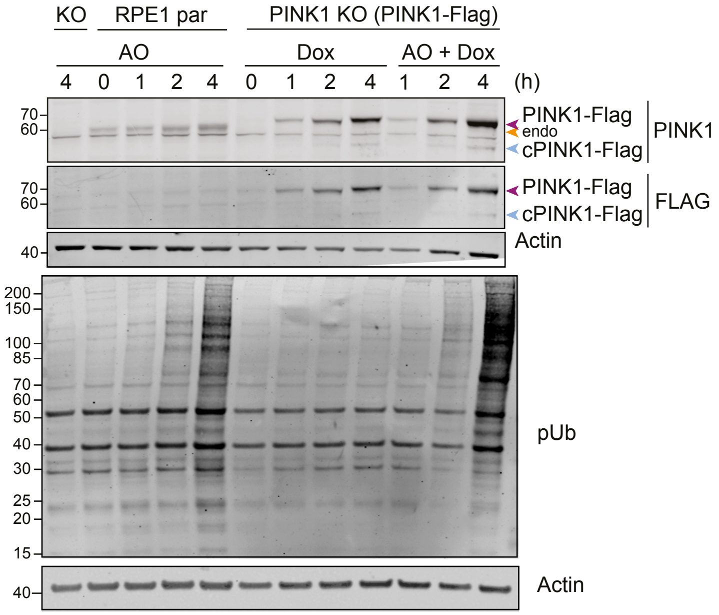


**Figure S2: Characterization of PINK1-Flag expression in hTERT-RPE1 PINK1 KO (PINK1-Flag) cells**

hTERT-RPE1 PINK1 KO cells (KO), parental hTERT-RPE1 (RPE1 par), and hTERT-RPE1 PINK1 KO (PINK1-Flag) cells were treated for the indicated time points ±Doxycycline (Dox, 0.1 µg/ml), ± Antimycin A and Oligomycin A (AO, 1 µM each) prior to lysis. Representative western blot of two independent experiments is shown, orange arrowhead indicates endogenous (endo) PINK1.

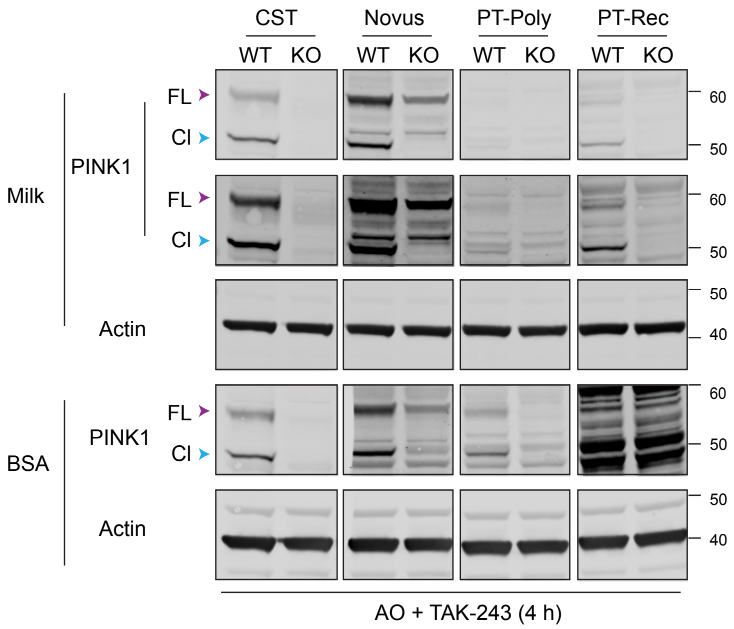


**Figure S3: Characterization of PINK1 antibodies**
hTERT-RPE1 parental (WT) and PINK1 KO cells were treated with ± Antimycin A and Oligomycin A (AO, 1 μM each), ± TAK-243 (1 μM) for 4h prior to lysis and probed with 4 different PINK1 antibodies using either 5% milk (Marvel) or 5% BSA as blocking and antibody dilution buffer (all diluted 1:1000). See Table S1 for further information on antibodies.

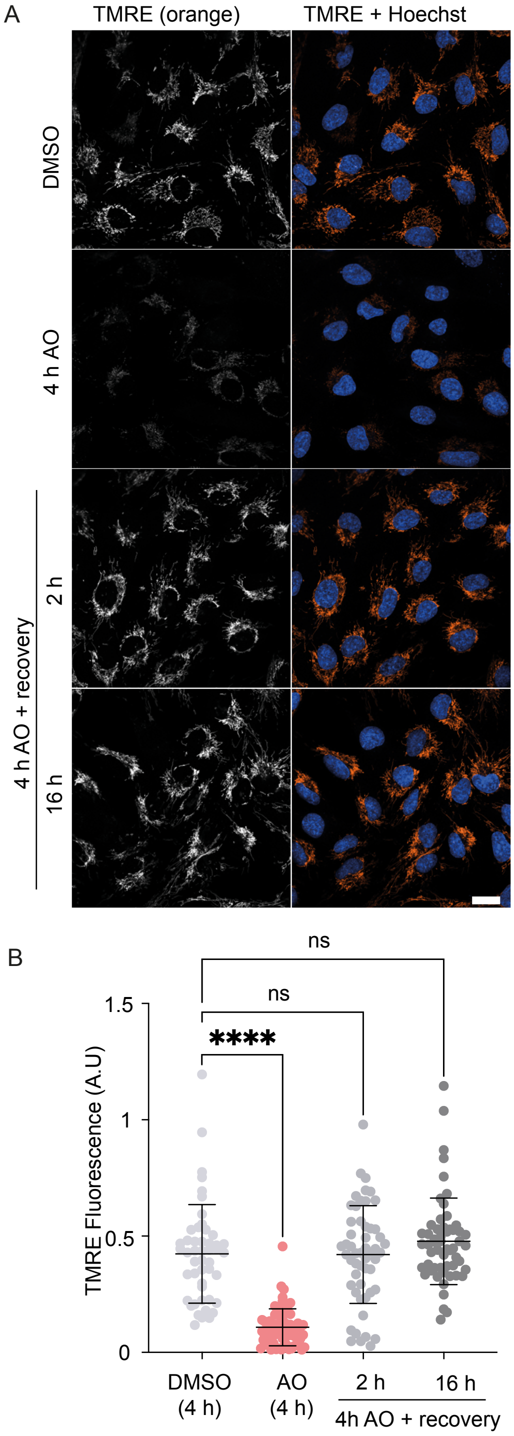


**Figure S4: Analysis of mitochondrial membrane potential**

**A.** hTERT-RPE1 cells were treated ± AO (1 μM) for 4 h and either immediately stained with TMRE (50 nM) and Hoechst 33342 (0.5 μg/ml) or first allowed to recover for indicated timepoints (recovery) prior to staining.

**B.** Quantification of TMRE fluorescence for each condition. Shown is the Mean Corrected Total Cell Fluorescence intensity for at least 49 cells per condition. Error bars indicate SD.

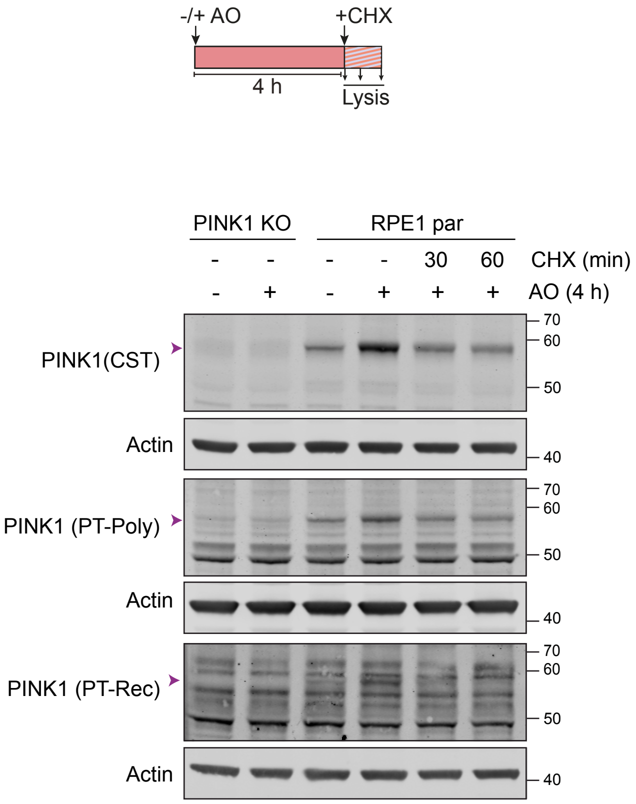


**Figure S5: Rapid decay of PINK1 under depolarizing conditions**

hTERT-RPE1 parental (par) and PINK1 KO cells were treated ± AO (1 μM each) for 4 h and either lysed immediately or treated in addition with cycloheximide (CHX; 100μg/ml) for 30 or 60 mins as in Figure 3A. Samples were probed with three different PINK1 antibodies. See Table S1 for further information on antibodies.

| Provider | Catalog number | Host species & Type | Immunogen |
|---|---|---|---|
| Cell Signalling Technologies (CST) | 6946 | Rabbit Monoclonal (D8G3) | synthetic peptide corresponding to residues surrounding Pro140 of human PINK1. |
| Novus Biologicals | BC100-494 | Rabbit Polyclonal | synthetic peptide made to the human PINK1 protein sequence (between residues 175-250) |
| Proteintech | 23274-1-AP | Rabbit Polyclonal | PINK1 fusion protein (GST-tagged, PINK1 aminoacids 336-581) |
| Proteintech | 81991-4-RR | Rabbit Recombinant | PINK1 fusion protein (GST-tagged, PINK1 aminoacids 336-581) |

**Table S1: Summary of PINK1 antibodies and associated immunogens**

Shown are the provider, Catalog number, host species and type, and Immunogen. Wherever not specified, the CST rabbit monoclonal PINK1 antibody D8G3 is used. The Novus antibody is used in Figure S3 and the Protein tech antibodies are used in Figures S3 and S5 alongside the CST antibody.
